# A Novel and Effective Method for Human Primary Skin Melanocytes and Metastatic Melanoma Cell Isolation

**DOI:** 10.3390/cancers13246244

**Published:** 2021-12-13

**Authors:** Aneta Ścieżyńska, Anna Sobiepanek, Patrycja D. Kowalska, Marta Soszyńska, Krzysztof Łuszczyński, Tomasz M. Grzywa, Natalia Krześniak, Agata Góźdź, Paweł K. Włodarski, Ryszard Galus, Tomasz Kobiela, Jacek Malejczyk

**Affiliations:** 1 Department of Histology and Embryology, Medical University of Warsaw, 02-004 Warsaw, Poland; asciezynska@wum.edu.pl (A.Ś.); marta.soszynska@onet.eu (M.S.); krzysztof.luszczynski99@gmail.com (K.Ł.); agata.gozdz@wum.edu.pl (A.G.); ryszard.galus@wum.edu.pl (R.G.); 2 Laboratory of Experimental Immunology, Military Institute of Hygiene and Epidemiology, 01-163 Warsaw, Poland; 3 Laboratory of Biomolecular Interactions Studies, Faculty of Chemistry, Warsaw University of Technology, 00-661 Warsaw, Poland; asobiepanek@ch.pw.edu.pl (A.S.); patrycja.kowalska3.dokt@pw.edu.pl (P.D.K.); kobiela@ch.pw.edu.pl (T.K.); 4 Polish Stem Cell Bank, 00-867 Warsaw, Poland; 5 Doctoral School, Medical University of Warsaw, 02-091 Warsaw, Poland; tomasz.grzywa@wum.edu.pl; 6 Centre for Preclinical Research, Department of Methodology, Medical University of Warsaw, 02-091 Warsaw, Poland; pawel.wlodarski@wum.edu.pl; 7 Department of Immunology, Medical University of Warsaw, 02-091 Warsaw, Poland; 8 Medical Centre of Postgraduate Education, Department of Plastic and Reconstructive Surgery, Prof. W. Orlowski Memorial Hospital, 00-416 Warsaw, Poland; natalia.krzesniak@wp.pl

**Keywords:** skin explants, melanocytes, melanoma, non-enzymatic cell isolation, spheroids

## Abstract

**Simple Summary:**

The present paper describes a simple, non-enzymatic and effective method of melanocyte or metastatic melanoma cell isolation from skin or lymph node explants, respectively. The method is based on selective harvesting of melanocytes or melanoma cells emigrating from the explants. Thus, isolated cells display specific phenotypical and functional features of melanocytes/melanoma cells such as tyrosinase and Melan-A expression and melanin production. Furthermore, melanocyte or melanoma cell cultures are not contaminated by keratinocytes and/or fibroblasts. The method appears to be a useful tool for studies on the biology of melanocytes and malignant melanoma.

**Abstract:**

The development of an effective method of melanocyte isolation and culture is necessary for basic and clinical studies concerning skin diseases, including skin pigmentation disorders and melanoma. In this paper, we describe a novel, non-enzymatic and effective method of skin melanocyte and metastatic melanoma cell isolation and culture (along with the spontaneous spheroid creation) from skin or lymph node explants. The method is based on the selective harvesting of melanocytes and melanoma cells emigrating from the cultured explants. Thereby, isolated cells retain their natural phenotypical features, such as expression of tyrosinase and Melan-A as well as melanin production and are not contaminated by keratinocytes and fibroblasts. Such melanocyte and melanoma cell cultures may be very useful for medical and cosmetology studies, including studies of antitumor therapies.

## 1. Introduction

Normal human skin melanocytes originate from the neural crest and represent about 3–7% of the epidermal cell population [1]. The role of cutaneous melanocytes is the production of melanin, a natural pigment responsible for skin coloration. Melanin is produced in the course of melanogenesis, a multistep process whereby tyrosine undergoes oxidation to 3,4-dihydrophenylalanine (DOPA) via the activity of tyrosinase. DOPA is subsequently polymerized to melanin [2]

Melanocyte dysfunction can result in the loss of pigmentation, and appearance of hypo- or hyperpigmented spots and may lead to the development of malignant melanoma [3]. According to WHO, 324,635 new cases of melanoma and 57,043 deaths related to this cancer were registered in 2020 around the world [4]. Melanoma is characterized by a very high risk of metastasis and displays a high mortality rate. Although great progress has recently been made in the treatment of melanoma (e.g., owing to the ipilimumab and nivolumab therapy), there is still a high proportion of poorly responding patients [5]. This is due to a high malignancy and heterogenicity of melanoma cells that make the available therapies not fully effective [6,7]. Thus, not surprisingly, melanoma is one of the most challenging cancers nowadays, in particular the cases of the refractory disease [8].

Melanoma and melanocyte research is mostly based on human cell culture models. These models include established human melanoma cell lines, where the most used are MeWo, A-375, SK-MEL-1, WM793, WM35, WM115, WM266-4, C32 and COLO 794. However, especially for the translational studies, there exists a need for the use of primary melanocyte and melanoma cell cultures. Such primary cultures may be very useful tools for, among other treatments, melanocyte transplantation treatment of vitiligo, or specific drug testing for personalized treatment of melanoma patients [9].

Several protocols for melanocyte and/or melanoma cell isolation and culture have been published [10,11], but these methods are based on the enzymatic separation of the epidermis from the dermis and the subsequent enzymatic release of single epidermal cells. A major limitation of these methods is the large size of skin sample needed to obtain a sufficient number of melanocytes [12]. Another difficulty is related to fibroblast and keratinocyte contamination. This may be overcome using the selection culture media supplemented with non-physiological substances that selectively promote the proliferation of melanocytes, e.g., 12-O-Tetradecanoylphorbol-13-acetate (TPA), phorbol-12-myristate-13-acetate (PMA), cholera toxin (CT) or 3-isobutyl-1-methylxanthine (IBMX) [13]. These compounds may significantly affect melanocyte biology, reduce the number of melanosomes and inhibit their terminal differentiation [14]. This limits the use of isolated and cultured melanocytes in some experimental and clinical approaches.

Considering the above, we focused on developing an alternative non-enzymatic method of skin melanocyte and metastatic melanoma cell isolation. This method is based on the selective harvesting of melanocytes and melanoma cells emigrating from skin and/or lymph node explants, respectively. The developed procedure is effective and avoids contamination by keratinocytes and fibroblasts. 

## 2. Materials and Methods

### 2.1. Collection of Tissue Samples

Normal human skin fragments were obtained from three female donors aged 35–50 years who underwent aesthetic breast reduction or aesthetic abdominoplasty at the Department of Plastic Surgery, Medical Centre of Postgraduate Education, Orlowski Memorial Hospital in Warsaw. Samples from two lymph nodes were obtained during surgical excision at the Maria Skłodowska-Curie Institute of Oncology, Warsaw, Poland from a 76-year-old male patient in whom recurrent metastatic malignant melanoma was confirmed clinically and histopathologically. 

Written informed consents were obtained from all participants. The study was approved and conducted according to strict guidelines of the local Ethical Committees (63/PB/2016 and KB/216/2017, respectively).

### 2.2. Cell Culture Media and Reagents

RPMI-1640, DMEM and MEME culture media, non-essential amino acids (NEAA), sodium pyruvate (SP) and penicillin-streptomycin antibiotic solution were purchased from Sigma Aldrich, Saint Louis, MO, USA. Medium 254, PMA-Free Human Melanocyte Growth Supplement-2 (HMGS-2), fetal bovine serum (FBS), phosphate-buffered saline (PBS) and 0.25% or 0.05% Trypsin-EDTA solution were obtained from Thermo Fisher Scientific Inc., Waltham, MA, USA.

### 2.3. Cell Lines

Lymph node-derived MeWo and WM266-4, skin-derived G-361 and amelanotic A-375 metastatic melanoma cell lines were obtained from American Tissue Culture Collection (ATCC, Manassas, VA, USA). Melanoma cell lines were cultured in RPMI-1640 medium supplemented with 10% FBS and 1% of the penicillin-streptomycin antibiotic solution. Spontaneously immortalized HaCaT keratinocytes (Deutsches Krebsforschungszentrum Stabsstelle Technologietransfer, Heidelberg, Germany) were cultured in DMEM supplemented with 10% FBS and 1% of the antibiotic solution. Immortalized CCD-1064Sk fibroblast cell line (ATCC) was cultured in MEME medium (Sigma) supplemented with 10% FBS, 1% NEAA, 1% SP and 1% penicillin-streptomycin antibiotic solution. All cells were cultured under standard conditions at 37 °C in 5% CO_2_ in the air and passaged with a Trypsin-EDTA solution (0.05% for melanoma cells and 0.25% for keratinocytes/fibroblasts) when necessary.

### 2.4. Isolation and Culture of Primary Melanocytes and Melanoma Cells

**Figure 1:**
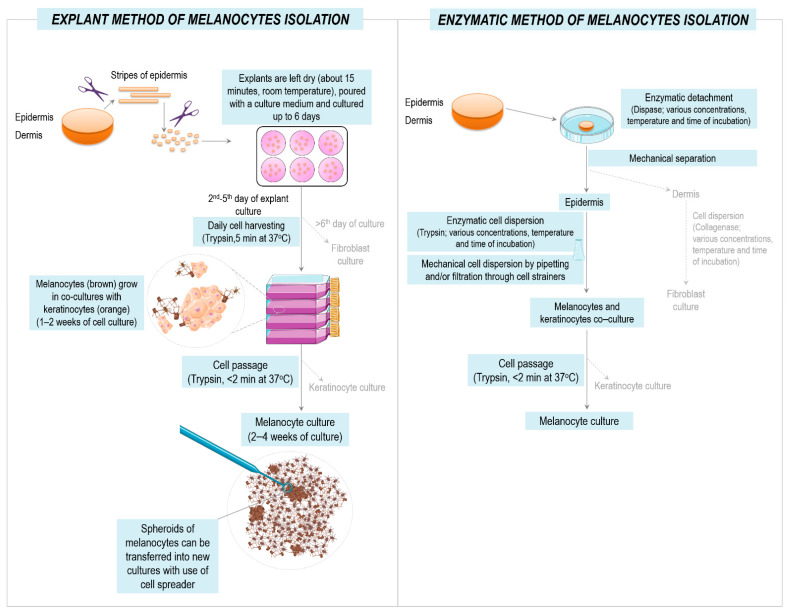
Scheme and timeline of procedures for melanocyte isolation from cultured tissue explants as compared with enzymatic cell isolation method. Graphics provided by smart.servier.com (4 December 2021).

Surgically collected tissue fragments (healthy skin or lymph node fragments) were transported to the laboratory on ice in a sterile ice-cold DMEM culture medium within 2 h of excision. The tissue fragments were then rinsed twice with an ice-cold PBS and transferred into a dedicated complete culture medium. The procedure of melanocyte isolation from the tissue explants and its comparison to the “classical” enzymatic method are summarized in Figure 1.

Thin strips of epidermis were mechanically separated from the dermis with scissors and subsequently cut into smaller, rectangular pieces of approximately 1 × 1 mm size. The lymph nodes were first cut into small pieces and next into rectangular pieces of approximately 1 × 1 mm size. Thereby, the prepared epidermal or lymph node explants were placed onto the wells of the tissue culture-treated six-well plates (cat. no. 703001; Nest Scientific Biotechnology, NJ, USA) and left dry for a maximum of 15 min. Afterwards, the wells with the normal skin explants were gently poured with 1.5 mL of DMEM culture medium supplemented with 20% FBS and 1% antibiotic solution, and the wells containing explants with the skin melanoma lesion or lymph nodes with metastatic melanoma were poured with the RPMI-1640 culture medium with 10% FBS and 1% antibiotic solution. The plates with explants were then incubated at 37 °C in the atmosphere of 5% CO_2_ in the air.

Melanocytes, together with keratinocytes emigrating from the normal skin explants, were harvested daily, starting from the second day of the explant incubation by serial trypsinization with 0.25% Trypsin-EDTA and seeded onto 25 cm^2^ tissue culture flasks (Nest Scientific Biotechnology) in Medium 254 culture medium supplemented with HMGS-2 and 1% antibiotic solution. The separation of melanocytes from explant-derived cells is described in the Section 3.

Melanoma cells emigrating from the lymph node explants were harvested for the first time after 5 days of incubation with the use of 0.05% Trypsin-EDTA and were cultured in 25 cm^2^ tissue culture flasks in RPMI 1640 culture medium supplemented with 10% FBS and 1% antibiotic solution.

Melanocytes and melanoma cells were routinely cultured under standard conditions at 37 °C in 5% CO_2_ in the air and were passaged with 0.05% Trypsin-EDTA when necessary.

### 2.5. Calculation of Melanoma Cell Population Doubling Time

In order to calculate the population doubling time (DT), the cells were seeded onto a 24-well plate in the number of 1 × 10^4^ cells/well. The cells were cultured under standard conditions, as described above, collected each day via trypsinization (from day 1 to 7) and counted in the Neubauer chamber. The DT calculation was based on the linear course of their growth curve. 

### 2.6. Morphometric Analysis of Melanoma Cells

Melanoma cell subcultures were seeded onto 24-well plates and cultured under standard conditions, as described above. After 48 h, the cells were washed with a PBS buffer, fixed in 4% formaldehyde and stained with 0.05% crystal violet solution in 1% methanol in water. Stained cells were washed with Milli-Q water, and cell images were captured under an inverted phase-contrast microscope at 10× magnification. Morphometric analysis of the cell shape was performed using ImageJ software (Rasband, W.S., ImageJ, U. S. National Institutes of Health, Bethesda, MD, USA).

### 2.7. Immunofluorescence Staining

Human primary melanocytes were cultured on 24-well cell culture plates under standard conditions, as described above. The cells emigrating from the skin explants were fixed in 4% formaldehyde, permeabilized in 0.1% Triton X-100 (Sigma) and subsequently incubated for 30 min at room temperature in a blocking solution of 3% bovine serum albumin (BSA). Then, cells were incubated for 1 h at RT with rabbit anti-tyrosinase (1:100, GTX16389; GeneTex, CA, USA) and mouse anti-collagen III (1:200, ab6310; Abcam, Cambridge, MA, USA) antibodies. For the detection of the primary antibodies, Alexa Fluor 594 goat anti-rabbit and Alexa Fluor 488 goat anti-mouse secondary antibodies (1:200, Thermo Fisher Scientific Inc.) were used. The nuclei were counterstained with DAPI (Thermo Fisher Scientific Inc.). 

### 2.8. RNA Isolation and cDNA Synthesis

The total RNA was isolated from at least 0.5 × 10^6^ cells with the use of AllPrep DNA/RNA KIT (Qiagen, Hilden, Germany). The quantity and purity of the isolated RNA were assessed by absorbance measurements at wavelengths of 260 and 280 nm using the NanoDrop 2000 spectrophotometer (Thermo Fisher Scientific), and 1 μg of the isolated RNA was reverse-transcribed into cDNA with the use of High-Capacity cDNA Reverse Transcription Kit (Thermo Fisher Scientific Inc., Waltham, MA, USA) with oligo dT primers according to the manufacturer’s instruction. 

### 2.9. Quantitative Reverse Transcription-Polymerase Chain Reaction (qRT-PCR)

The qRT-PCR was performed with the use of the SensiFAST™ Probe Lo-ROX Kit (Bioline Meridian Life Science, Memphis, TN, USA) on the Applied Biosystems™ 7500 Real-Time PCR System (Thermo Fisher Scientific Inc.). The expression of tyrosinase (*TYR*), *MLANA* and *COL1A2* mRNA was evaluated using Hs00165976_m1, Hs00194133_m1 and Hs01028956_m1 TaqMan probes (Thermo Fisher Scientific Inc.), respectively. The *TBP* (Hs00427620_m1) and *GAPDH* (Hs02758991_g1) probes served as an endogenous control. The relative mRNA expression of the investigated genes was calculated using the 2^−∆∆Ct^ method. 

### 2.10. Flow Cytometry Analysis

The cells were harvested by trypsinization, stained with Zombie NIR™ (BioLegend) and blocked on ice with Human TruStain FcX™ (Fc Receptor Blocking Solution) (Biolegend) in a FACS buffer (PBS; 1% BSA, 0.01% sodium azide). Following 30 min of incubation on ice with anti-MELAN-A antibody (Thermo Fisher Scientific Inc.), the cells were washed three times in a FACS buffer and were incubated for 30 min with Alexa Fluor 555-conjugated anti-mouse antibody. After washing in a FACS buffer, the cells were immediately analyzed on a BD LSRFortessa™ X-20 flow cytometry analyzer with FACSDiva software (BD Biosciences, San. Diego, CA, USA). The gates for MELAN-A-positive cells were adjusted based on the control cells stained with only secondary antibody. The obtained data were analyzed with the use of the Flow Jo v10.6.1 software (TreeStar, Ashland, OR, USA).

## 3. Results

### 3.1. Isolation and Culture of Primary Human Melanocytes from Skin Explants

**Figure 2:**
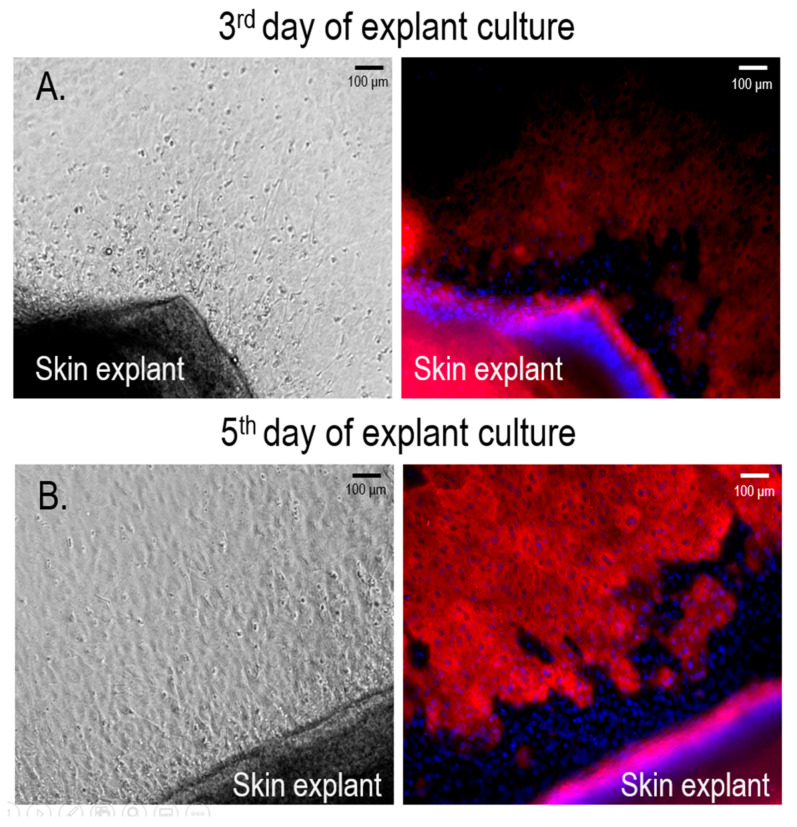
Expression of tyrosinase by the cells emigrating from skin explant after 3 (**A**) and 5 (**B**) days of explant culture, as revealed by immunofluorescence microscopy. The cells were stained with the primary anti-tyrosinase antibody, followed by the secondary antibody labeled with Alexa Fluor 594 (red fluorescence). The nuclei were stained with DAPI (blue fluorescence). See the Section 2 and Figure 1 for details of the procedure.

Microscopic observation of skin explants revealed the emigration of cells usually starting from the second day of culture. On the third day, the migrating cells formed pavement-like rings surrounding the skin explants (Figure 2A). Among the emigrating cells, single cells with dendritic processes and well-defined borders were observed. Immunofluorescent staining with anti-tyrosinase antibodies revealed a positive reaction in cells in the front of the migration ring. More advanced cell migration was observed from the fifth day, from which point nearly all emigrating cells displayed strong tyrosinase immunostaining (Figure 2B).

**Figure 3:**
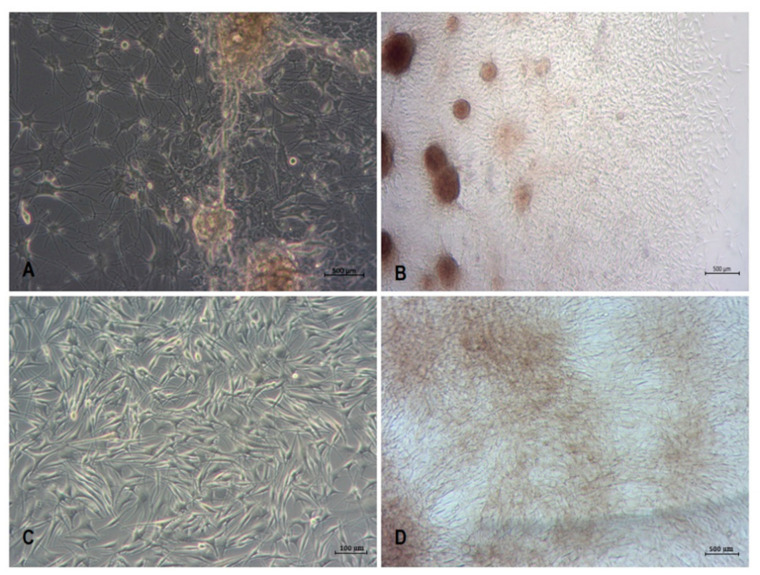
Stages of primary melanocyte culture. (**A**) One-week-old co-culture of melanocytes and keratinocytes following their harvesting on the second and fifth days of skin explant culture. (**B**) Two-week-old melanocyte monoculture originating from two-week-old melanocyte and keratinocyte co-culture. Please note the appearance of pigmented spheroids in the monoculture of melanocytes. (**C**) Subconfluent melanocyte culture originating from the spheroids. (**D**) Pigmented melanocyte culture originating from the spheroids. See the Section 2 and Figure 1 for details of the procedure.

**Figure 4:**
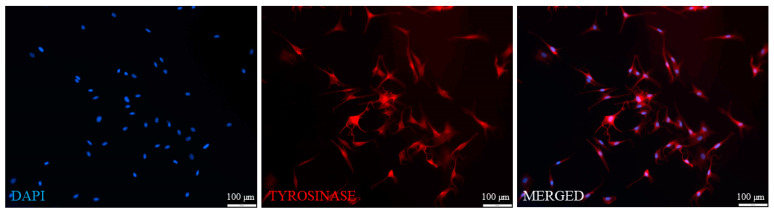
Expression of tyrosinase by the cultured melanocytes originating from the two-week-old melanocyte and keratinocyte co-culture, as revealed by immunofluorescence microscopy. The cells were stained with the primary anti-tyrosinase antibody, followed by the secondary antibody labeled with Alexa Fluor 594 (red fluorescence). The nuclei were stained with DAPI (blue fluorescence). See the Section 2 and Figure 1 for details of the procedure.

Outgrowing cells were gently detached from the skin explants after a short trypsinization and seeded onto 5 mL flasks upon further culture. Detachment of the outgrowing cells and their passaging to separate culture flasks was performed every consecutive day until the fifth day of explant culture. Thereby, the obtained cultures consisted of colonies of keratinocyte-like cells and cells displaying dendritic-like shape (Figure 3A). After two weeks of culture, the dendritic-like cells formed colonies. The growth of some of these colonies resulted in the formation of pigmented spheroids (Figure 3B). The first passage of these primary cultures using short, up-to-2-min trypsinization resulted in a selective separation of dendritic-like cells (Figure 3C). With the following culture, some of these cells displayed what appeared to be a brown-black pigmentation (Figure 3D). Immunofluorescent staining of the first passage cultures with specific anti-tyrosinase antibodies revealed a positive reaction for tyrosine in almost 100% of the cultured cells (Figure 4).

**Figure 5:**
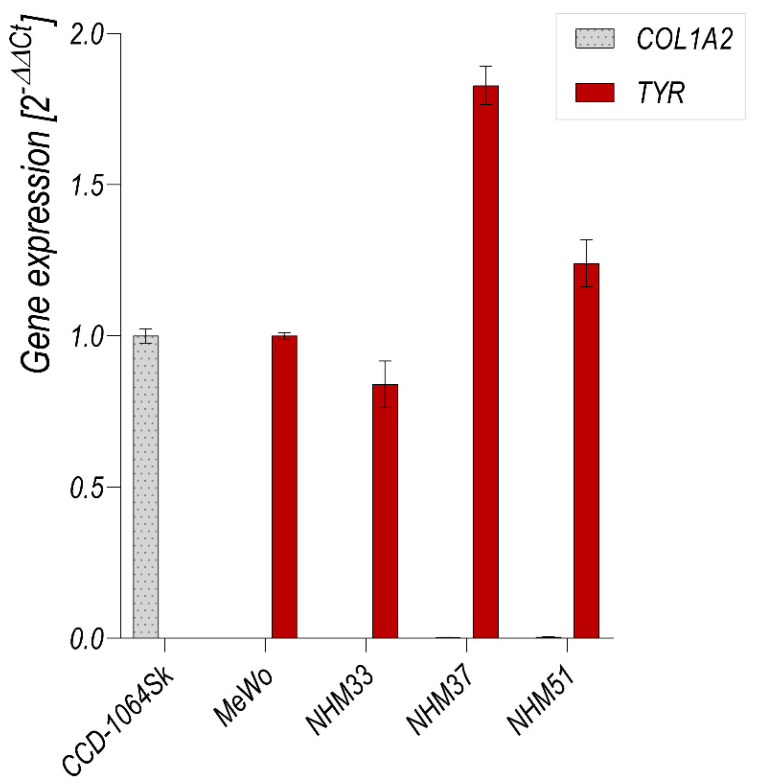
qRT-PCR analysis of the expression of *TYR* and *COL1A2* mRNA in the human melanocyte cultures (NHM33, NHM37 and NHM51) obtained after the first passage of cells originating from the spheroids, CCD-1064Sk fibroblast cell line and MeWo melanoma cell line. The *COL1A2* expression was normalized to the expression of CCD-1064Sk, and that of *TYR* was normalized to the expression of MeWo.

The expression of tyrosinase (*TYR*) mRNA by the primary melanocyte cultures was further confirmed by qRT-PCR reaction (Figure 5). As can be seen, these cells expressed *TYR* mRNA at a level similar to the MeWo melanoma cell line. On the other hand, *TYR* mRNA was undetected in the CCD-1064Sk skin fibroblast cell line. The CCD-1064Sk fibroblasts expressed collagen (*COL1A2*) mRNA, whereas *COL1A2* mRNA was undetected in both primary melanocytes and MeWo cells.

### 3.2. Isolation and Culture of Human Melanoma Cells from Explants with Melanoma Metastases

**Figure 6:**
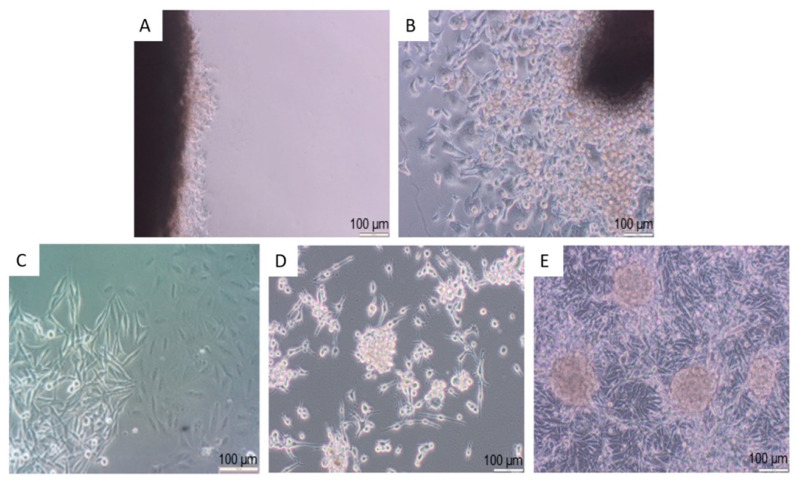
Stages of the patient-derived melanoma cell culture. Cells emigrating from lymph node explant on the first (**A**) and fifth (**B**) days of culture. (**C**) Three-day-old culture of melanoma cells harvested 5 days after lymph node explant inoculation. (**D**) Spheroids’ formation in two-week-old culture of melanoma cells harvested 5 days after lymph node explant inoculation. (**E**) Three-week-old culture of melanoma cells originating from a single spheroid.

Microscopic observation of the explants from a patient with metastatic melanoma showed emigration of cells as early as in a one-day culture (Figure 6A). During the following days, the expansion of emigrating cells was considerably increased (Figure 6B). These emigrating cells displayed elongated phenotype with processes; some of them also showed lymphoid-like phenotype. 

**Figure 7:**
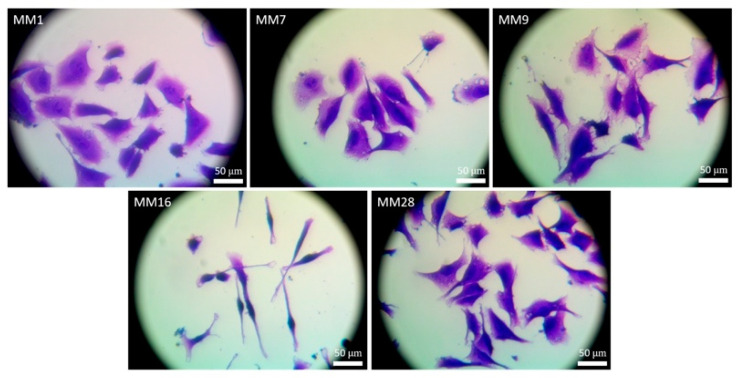
Phenotypic appearance of melanoma cell fractions propagated from the different spheroids obtained from melanoma cell culture harvested 5 days after lymph node explant inoculation (MM1, MM7, MM9, MM16 and MM28). The cells were fixed and stained with crystal violet.

**Figure 8:**
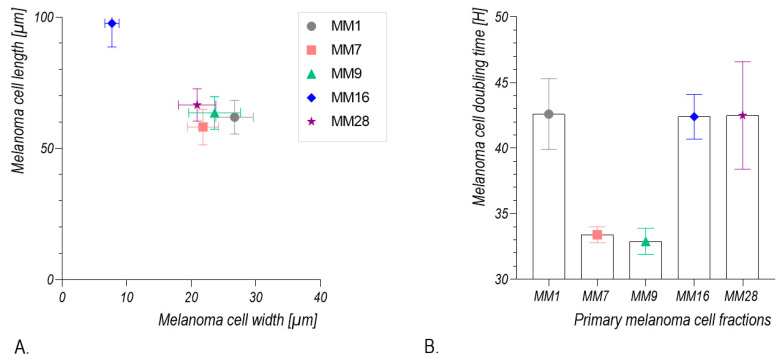
Morphometric analysis and proliferative potential of the melanoma cell fractions from different spheroids (MM1, MM7, MM9, MM16 and MM28) obtained from melanoma cell culture harvested 5 days after lymph node explant inoculation. (**A**) Cell shape analysis of melanoma cells. **(B)** Population doubling time of melanoma cells from different spheroids.

The emigrating cells were harvested by trypsinization on the fifth day of explant culture. Thereby, the obtained cells formed colonies of different sizes and morphology (Figure 6C). Some cells spontaneously formed spheroid-like colonies (Figure 6D,E). In some spheroid-like colonies, the cells displayed different morphological features. These spheroids were mechanically separated and placed in separate cultures. Subculture of these separated cells resulted in the isolation of five distinct melanoma cell fractions designated as MM1, MM7, MM9, MM16 and MM28 cells (Figure 7). As can be seen, the melanoma cells of these distinct fractions displayed different morphological features. MM16 cells had a spindle shape, whereas other melanoma cell fractions (MM1, MM7, MM9 and MM28) showed epithelioid morphology. MM16 cells were remarkably longer but narrower than the cells from the other fractions (Figure 8A).

In long-term cultures, the MM7, MM9, MM16 and MM28 cells formed spheroid-like structures, while spheroids were not formed by the MM1 cells. An analysis of melanoma cell proliferation revealed that the population doubling time of MM7 and MM9 cells was considerably shorter when compared with the other three cell fractions (Figure 8B). 

**Figure 9:**
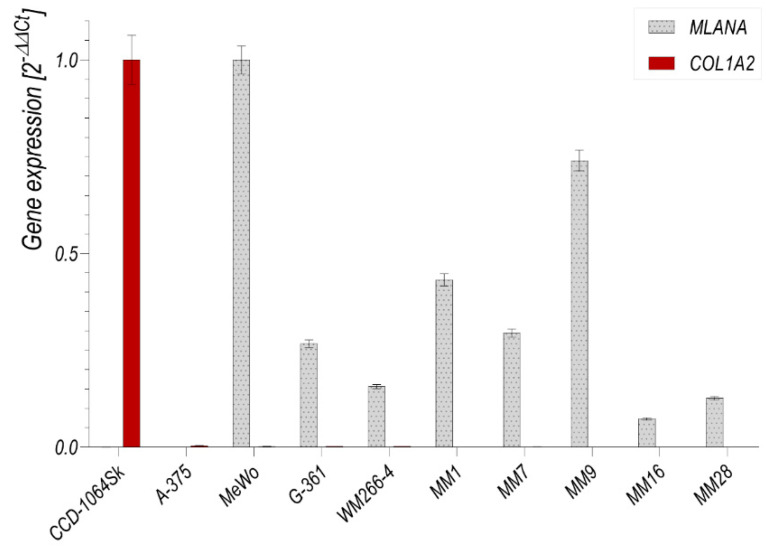
qRT-PCR analysis of *MLANA* and *COL1A2* mRNA expression in the melanoma cell fractions from different spheroids (MM1, MM7, MM9, MM16 and MM28) obtained from melanoma cell culture harvested 5 days after lymph node explant inoculation; CCD-1064Sk fibroblast cell line; and A-375, WM266-4, G-361 and MeWo metastatic melanoma cell lines. *COL1A2* and *MLANA* expression in the studied cells was normalized to CCD-1064Sk and MeWo cells, respectively.

All cell fractions, as well as MeWo, G-361 and WM266-4 melanoma cell lines, expressed mRNA for the *MLANA,* gene as revealed by qRT-PCR analysis (Figure 9). The expression of *MLANA* was not found in the amelanotic A-375 melanoma cell line and the CCD-1064Sk skin fibroblast cell line. CCD-1064Sk skin fibroblasts expressed the *COL1A2* gene. The expression of *COL1A2* was not found in all melanoma cell fractions, or in any melanoma cell lines that were studied. 

### 3.3. Flow Cytometry Analysis of Isolated Melanocytes and Melanoma Cells

**Figure 10:**
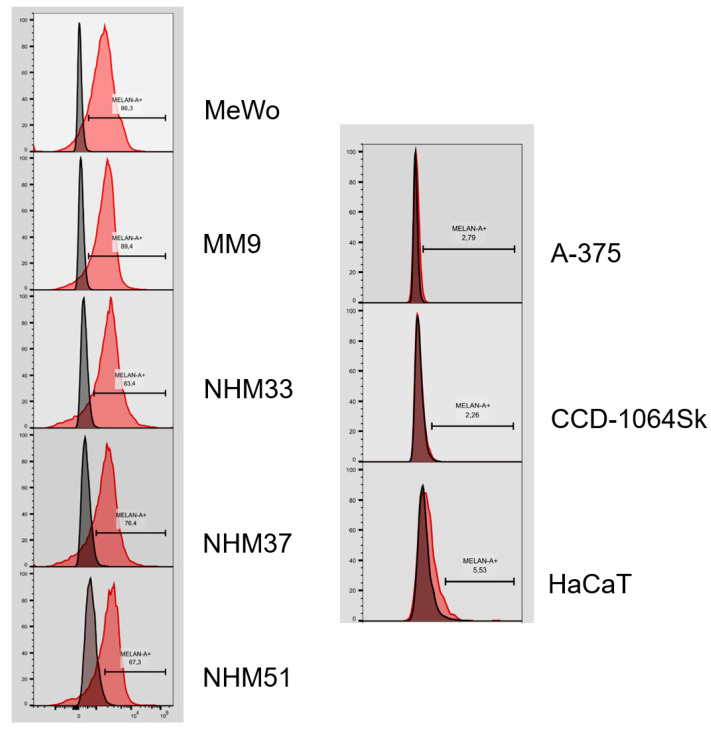
Flow cytometry analysis of Melan-A expression in the isolated melanocytes (NHM33, NHM37 and NHM51) and melanoma cell fraction (MM9) as compared with MeWo melanoma, amelanotic A-375 melanoma, CCD-1064Sk fibroblast and HaCaT keratinocyte cell lines.

Flow cytometry analysis confirmed that the isolated melanocyte cell lines (NHM33, NHM37 and NHM51) and MM9 melanoma cell fraction expressed Melan-A, an antigen expressed by melanocytes and melanoma cells (Figure 10). As is shown, over 75% of the isolated melanocytes and melanoma cells and 86.3% of MeWo melanoma cells were Melan-A-positive. The percentages of Melan-A-positive cells in A-375 amelanotic melanoma, CCD-1064Sk fibroblast and HaCaT keratinocyte cell lines were not higher than 6%.

## 4. Discussion

In this study, we describe a new, simple and effective method for skin melanocyte and metastatic melanoma cell isolation and culture. The method does not include enzymatic tissue dispersion and is based on the specific harvesting of melanocytes or metastatic melanoma cells sequentially emigrating from skin or lymph node explants. This is an important advantage of our method since trypsin, collagenase or dispase treatment (prior to initial epidermis and dermis separation, as well as subsequent epidermis disintegration) may result in a substantial reduction in the viability of keratinocyte and melanocytes [15,16]. 

Our observations confirm that cell populations emigrating from skin explants during the first few days of culture consist of both melanocytes and keratinocytes [17]. The harvesting of both keratinocytes and melanocytes commonly outgrowing from epidermis explants during the first few days of incubation may be advantageous to melanocyte growth since keratinocytes secrete substances that support melanocyte growth [18]. Furthermore, keratinocytes may be easily removed from the culture during the first passage as they require a substantially longer time for trypsinization than melanocytes, which detach within 2 min [19]. 

We did not observe an emigration of fibroblasts within 5 days of the explant incubation, and this is in agreement with a report by Guo et al. [20], who noticed the outgrowth of fibroblasts from skin explants after just 7 days of culture. Analogical observation of the isolation of metastatic melanoma cells from lymph node explants was conducted, and melanoma cells appeared to be a major population of emigrating cells. Thus, it may be concluded that the harvesting of emigrating cells within 5 consecutive days avoids contamination of the primary cell cultures with fibroblasts. This is important since contaminating fibroblasts rapidly overgrow melanocyte and keratinocyte cultures, and their elimination requires treatment with selective agents, e.g., geneticin [21].

The effectiveness and usefulness of the method presented above were further confirmed by the analysis of the phenotype of the melanocyte and melanoma cell cultures that we obtained. We confirmed that the cultured melanocytes or melanoma cells displayed the tyrosinase or Melan-A protein and mRNA expression. The expression of those markers was found in almost all of the examined cells, as judged by immunofluorescence and cell cytometry with specific antibodies. Analysis of collagen expression further confirmed that the cultures were not contaminated by fibroblasts.

Interestingly, the prolonged culture of primary melanocytes or melanoma cells resulted in the spontaneous formation of spheroid-like structures. It has been previously reported that melanocytes form three-dimensional spheroids when cultured in serum-deprived conditions on a surface coated with chitosan. They also displayed the ability to form spheroids after repetitive long-term trypsinization [22]. It is noteworthy that melanocytes cultured in spheroids have higher viability and functional activity than melanocytes cultured in monolayers [3,22]. Furthermore, melanocytes isolated from the spheroids possess morphological features of melanocyte stem cells, as they displayed high proliferative potential [10,22]. We observed that melanoma cells originating from spheroid-like structures also had a relatively high proliferation rate.

The major function of melanocytes is melanin production, which they owe to tyrosinase activity. The production of melanin may depend on the culture conditions (such as 2D culture or medium type) and its duration [23]. It has been reported that melanocytes lose the ability to synthesize melanin in a monolayer culture [3]. However, we observed that melanocytes and melanoma cells retained the ability to produce melanin, even when growing in a monolayer culture. The melanocytes and melanoma cells characterized in this study expressed tyrosinase as well as Melan-A protein, which plays a crucial role in melanosome biogenesis and is considered a marker of melanocytes and melanomas [24]. This strengthens our argument that the proposed method of melanocyte and melanoma cell isolation and culture allows them to protect their phenotype.

In our study, the subculture of cells from spheroid-like aggregates resulted in establishing five fractions of melanoma cells characterized by different morphologies. These cell fractions displayed various levels of *MLANA* gene expression and proliferative potential. The proliferation of melanoma cells may depend on different factors such as culture conditions and the composition of the culture medium [25]. Generally, the population doubling time for metastatic melanoma cells was reported to be in the range of 6–29 h [26]. The population doubling time of melanoma cells isolated and cultured in our study ranged from 33 to 42 h according to the cell fraction. The proliferation potential was associated with neither cell morphology nor *MLANA* gene expression.

## 5. Conclusions

The method of melanocyte and melanoma cell isolation and culture presented appears to be simple and effective. Cultures of melanocytes and melanoma cells obtained by this method are not contaminated by other cell types, and the cells retain their natural phenotypical features such as expression of tyrosine and Melan-A, as well as melanin production. Such melanocyte and melanoma cell cultures may be very useful for medical and cosmetology studies, including studies on new antitumor therapies.

## Data Availability

The data presented in this study are available on request from the corresponding author.
